# HIF-1α restricts NF-κB-dependent gene expression to control innate immunity signals

**DOI:** 10.1242/dmm.017285

**Published:** 2014-12-15

**Authors:** Daniel Bandarra, John Biddlestone, Sharon Mudie, H.-Arno J. Müller, Sonia Rocha

**Affiliations:** 1 Centre for Gene Regulation and Expression, College of Life Sciences, University of Dundee, Dow Street, DD1 5EH, UK.; 2 Division of Cell and Developmental Biology, College of Life Sciences, University of Dundee, Dow Street, DD1 5EH, UK.

**Keywords:** HIF-1, IKK, NF-κB, Hypoxia, Inflammation, *Drosophila*

## Abstract

Hypoxia and inflammation are intimately linked. It is known that nuclear factor κB (NF-κB) regulates the hypoxia-inducible factor (HIF) system, but little is known about how HIF regulates NF-κB. Here, we show that HIF-1α represses NF-κB-dependent gene expression. HIF-1α depletion results in increased NF-κB transcriptional activity both in mammalian cells and in the model organism *Drosophila melanogaster*. HIF-1α depletion enhances the NF-κB response, and this required not only the TAK-IKK complex, but also CDK6. Loss of HIF-1α results in an increased angiogenic response in mammalian cancer cells and increased mortality in *Drosophila* following infection. These results indicate that HIF-1α is required to restrain the NF-κB response, and thus prevents excessive and damaging pro-inflammatory responses.

## INTRODUCTION

In response to a drop in oxygen (hypoxia), the cell orchestrates a variety of coordinated responses in order to restore oxygen homeostasis. Although the physiological responses to hypoxia are well established, the molecular processes activated within the cells are not completely understood. One major contribution to this field was the discovery of a transcription factor that responds to hypoxia, hypoxia-inducible factor (HIF) (Semenza and Wang, 1992). HIF was first identified in 1995 together with the hypoxia response element (HRE, 5′-RCGTG-3′) of the erythropoietin gene (*EPO*) (Wang and Semenza, 1995). Further studies revealed that HIF is actually a heterodimeric complex composed of an α and a β subunit, which exist as a series of isoforms: 1α, 2α and 3α (Rocha, 2007). Even though the expression of the HIF-1β gene and protein is not dependent upon oxygen changes, HIF-α subunits are extremely labile at normal oxygen levels (Kenneth and Rocha, 2008).

The activity of the complex HIF-1α–HIF-1β is determined by the stabilisation of the α subunit during hypoxia. In the presence of oxygen (normoxia), HIF-α is regulated by a class of dioxygenases called prolyl-hydroxylases (PHD1, PHD2 and PHD3) (Kaelin, 2005). The hydroxylation of specific prolyl residues promotes the interaction of HIF with the von Hippel-Lindau E3 ligase (VHL) and, hence, proteasomal-mediated proteolysis (Maynard et al., 2003). Thus, when oxygen levels are reduced, or any of the PHD co-factors are unavailable, there is an increase of HIF-α subunit levels resulting from the inability of VHL to efficiently bind to HIF-α.

To date, more than 100 HIF target genes have been identified, which are involved in key cellular processes, such as angiogenesis, glucose and energy metabolism, and cell growth and apoptosis (Schofield and Ratcliffe, 2004).

Although most knowledge regarding HIF has been derived from studies following hypoxic stress, HIF-α stabilisation has also been found in non-hypoxic settings, such as relatively well-oxygenated regions of tumours, and in diseases such as rheumatoid arthritis and diabetes (Catrina et al., 2004; Taylor and Sivakumar, 2005). Recent studies have demonstrated HIF-1α to be regulated by the major inflammation-responsive transcription factor, nuclear factor κB (NF-κB) (Bonello et al., 2007; Rius et al., 2008; van Uden et al., 2008). In the absence of NF-κB, the HIF-1α gene is not efficiently transcribed and, hence, no stabilisation or activity is seen, even after prolonged exposure to hypoxia (Kenneth et al., 2009; van Uden et al., 2008).

NF-κB is the collective name for a family of transcription factors that includes RelA (p65), RelB, c-Rel, NF-κB1 (p105/p50) and NF-κB2 (p100/p52). NF-κB is normally held inactive in the cytoplasm by the IκB family of inhibitory proteins. After an activating stimulus, such as the inflammatory tumour necrosis factor α (TNF-α) cytokine, IκB is phosphorylated by the IKK complex, which targets IκB for degradation. Degradation of IκB results in the release of the NF-κB dimer and its translocation into the nucleus (Hayden and Ghosh, 2008; Perkins, 2006; Perkins and Gilmore, 2006).

NF-κB has hundreds of direct targets, including genes that are involved in inflammation, cell cycle and apoptosis (Perkins, 2012). The specificity and complexity of the NF-κB response comes from the possible homo- and heterodimer formation between subunits, the recruitment of co-activator and co-repressor complexes to particular promoters, as well as post-translational modifications of the NF-κB subunits themselves (Perkins, 2012). Aberrantly active NF-κB has been associated with a number of human diseases, stimulating the pharmaceutical industry’s interest in finding potential approaches to inhibit NF-κB (Karin et al., 2004).

The finding that NF-κB activation results in HIF stabilisation and activity suggests a functional involvement of the HIF transcription factor in processes where NF-κB is involved, in particular in response to infection and inflammation. In the present study, we show that HIF-1α plays an important role in regulating the NF-κB pathway. Surprisingly, HIF-1α acts to restrict NF-κB transcriptional activity, in mammalian cells and in *Drosophila*. Depletion of HIF-1α results in increased levels of specific NF-κB targets, such as p100 and interleukin 8 (IL-8), by a mechanism that is dependent on TAK and IKK, and CDK6. Functionally, depletion of HIF-1α results in increased pro-angiogenic IL-8 production and increased angiogenesis in an *in vitro* model. Deletion of the HIF-1α homologue in *Drosophila*, Sima, results in hypersensitivity to infection due to deregulated NF-κB. These results delineate, for the first time, the contribution of HIF-1α towards the NF-κB pathway and demonstrate the importance of HIF-1α presence for the control of the inflammatory response *in vivo*.

**TRANSLATIONAL IMPACT**

**Clinical issue**

Low oxygen (hypoxia) and inflammation are intimately linked and often present in many human diseases. The hypoxia-inducible factor (HIF) system plays an important role in the response to low oxygen and comprises several factors and subunits, including HIF-1α. HIF-α is involved in key cellular processes not only during hypoxia but also in non-hypoxic and inflammatory conditions, such as rheumatoid arthritis and diabetes. It is known that the transcription factor nuclear factor κB (NF-κB; which is associated with inflammation) regulates HIF-1α, but little is known about how the HIF system regulates NF-κB.

**Results**

In this study, the authors use human cell lines and *Drosophila melanogaster* to investigate how HIF-1α regulates NF-κB activity, and they show that HIF-1α represses NF-κB-dependent gene expression. In particular, HIF-1α depletion results in increased NF-κB transcriptional activity, both in mammalian cells and in a whole organism (*Drosophila*). HIF-1α depletion enhances the NF-κB response and this requires the involvement of other factors, including the TAK-IKK complex and cyclin-dependent kinase 6 (CDK6). Loss of HIF-1α results in an increased angiogenic response in human cancer cells and increased mortality in *Drosophila* following infection.

**Implications and future directions**

These results indicate that HIF-1α is required to restrain the NF-κB response and, thus, it is important to prevent excessive and damaging pro-inflammatory responses during infection and inflammation. Furthermore, this study suggests that targeting HIF-1α in disease might result in undesirable secondary effects, with increased angiogenesis and excessive inflammation.

## RESULTS

### HIF levels and activity are induced following infection in *Drosophila*

**Fig. 1.**
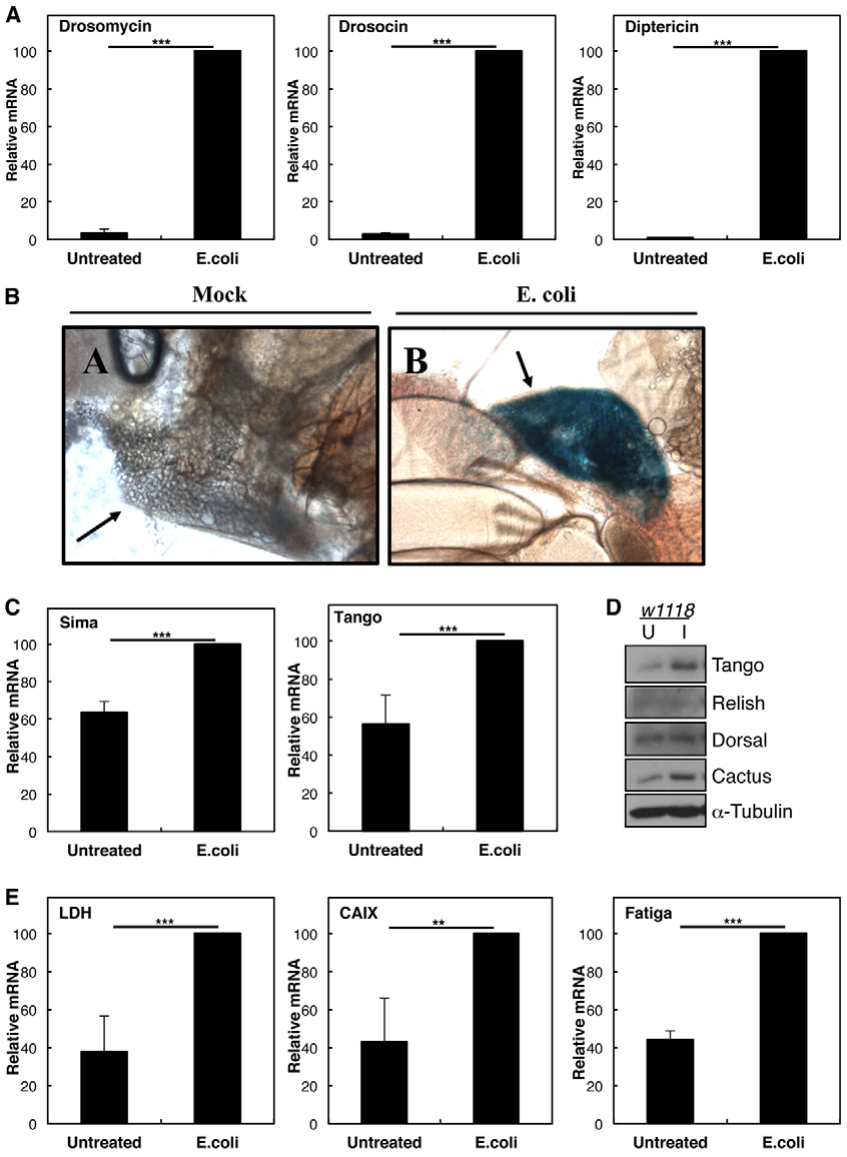
**NF-κB-mediated regulation of the HIF pathway is conserved in *Drosophila*.** (A) Third instar larvae were infected with *E. coli* and left for 2 h at 25°C before lysis. Total RNA was extracted from the whole body of control larvae, and RT-qPCR was performed to determine the levels of *drosomycin*, *drosocin* and *diptericin* in untreated or infected flies. (B) Fat body tissue sections from young adult flies (arrows) reported by β-gal staining of *diptericin-lacz*. Panel A, mock infection. Panel B, *E. coli* infection. (C,E) As A, but levels of *sima*, *tango* (C), *ldh*, *fatiga and caix* (E) were measured in untreated or infected flies. (D) Protein was extracted from the whole body of larvae and analysed by western blotting using the indicated antibodies. The graph shows the relative levels of specific mRNA transcripts normalised to actin mRNA levels, quantified relative to infected flies. The means+s.d. were determined from three independent experiments. Student’s *t*-test analysis was performed *P≤0.05, **P≤0.01, ***P≤0.001.

We have previously demonstrated that, in mammalian cancer cell lines, TNF-α-induced NF-κB can increase HIF-1α mRNA, protein and activity levels, leading to transactivation of target genes in normoxia (van Uden et al., 2008; van Uden et al., 2011). However, whether this induction occurs in the context of a whole organism, and its physiological role, remain unknown. To address this question, we have chosen the model organism *Drosophila*, in which both the NF-κB and HIF pathways are conserved, and their biological roles are well established (Arquier et al., 2006; Hetru and Hoffmann, 2009; van Uden et al., 2011). In *Drosophila*, NF-κB is activated by infection and plays a crucial role in the immune response. Third instar larvae were infected with *Escherichia coli* (*E. coli*), and the mRNA levels of *drosomycin*, a peptide induced in bacterial infections, were measured by using real-time quantitative PCR (RT-qPCR) as a control for infection (Fig. 1A). As observed in Fig. 1A, larvae were successfully infected because a significant increase of *drosomycin* was found when compared with that of non-infected animals. Additional NF-κB targets, *drosocin* and *diptericin*, were also induced under these conditions (Fig. 1A). We could also observe induction of a *lacz-diptericin* reporter in the fat body of young adult flies following infection with *E. coli* (Fig. 1B). To test whether the HIF subunits were responsive to infection *in vivo*, we analysed the levels of *Drosophila* HIF-α and HIF-β mRNA (*sima* and *tango*, respectively) (Fig. 1C). In response to *E. coli* infection, we measured a statistically significant increase in both the levels of *sima* and *tango* mRNA (Fig. 1C). We also detected an increase in Tango protein levels by western blotting (Fig. 1D). Unfortunately, we could not analyse the levels of Sima because there are no commercially available antibodies. Nevertheless, we determined the relevance of the mRNA increase observed for the HIF subunits by measuring whether HIF-dependent genes were transcriptionally induced in response to infection (Fig. 1E). As can be seen in Fig. 1E, the expression of several of HIF-dependent genes, such as *ldh*, *caix* and *fatiga*, were all significantly induced following infection, indicating that the HIF pathway had been activated.

### HIF activation following infection in *Drosophila* is dependent upon the NF-κB pathway

**Fig. 2.**
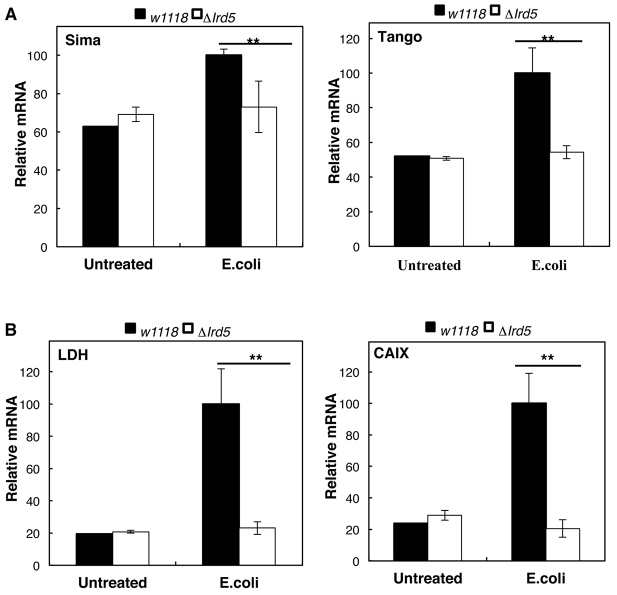
**IKK-dependence of HIF activation by inflammation is conserved *in vivo*.** Third instar larvae were infected with *E. coli* and left for 2 h at 25°C before to lysis. Total RNA was extracted from the whole body of control (*w1118*) or IKK loss-of-function (Δ*Ird5*) larvae, and RT-qPCR was performed to determine the levels of *sima* and *tango* (A), and *ldh and caix* (B) in untreated or infected flies. The graph shows the relative levels of specific mRNA transcripts normalized to *actin* mRNA levels. The means+s.d. were determined from three independent experiments. Student’s *t*-test analysis was performed *P≤0.05, **P≤0.01, ***P≤0.001. See also supplementary material Fig. S1.

Given that, in mammalian cells, inflammation-induced HIF is dependent upon the upstream kinase that activates NF-κB (IKK) (van Uden et al., 2008), we asked whether this mechanism was conserved in *Drosophila*. To answer this question, we used a loss-of-function allele for *ird5*, *ird5*^1^, the IKK homologue in *Drosophila* (Lu et al., 2001). Wild-type and *ird5*^1^ homozygous mutant flies were infected with *E. coli*, and the levels and activity of HIF were analysed (Fig. 2A). Our analysis demonstrated that, as expected, the levels of the NF-κB target *drosomycin* were no longer induced upon infection, when *ird5* was absent (supplementary material Fig. S1). Consistent with our previous observations in mammalian systems, in the absence of *ird5* (IKK), both *sima* and *tango* levels failed to be induced following infection (Fig. 2A). Notably, in the IKK loss-of-function flies, the levels of the HIF-dependent targets *ldh* and *caix* were no longer increased under conditions of infection (Fig. 2B). These data indicate that the IKK dependence of HIF activation through inflammation *in vivo* is evolutionary conserved.

### The role of HIF in the immune response of *Drosophila*

**Fig. 3.**
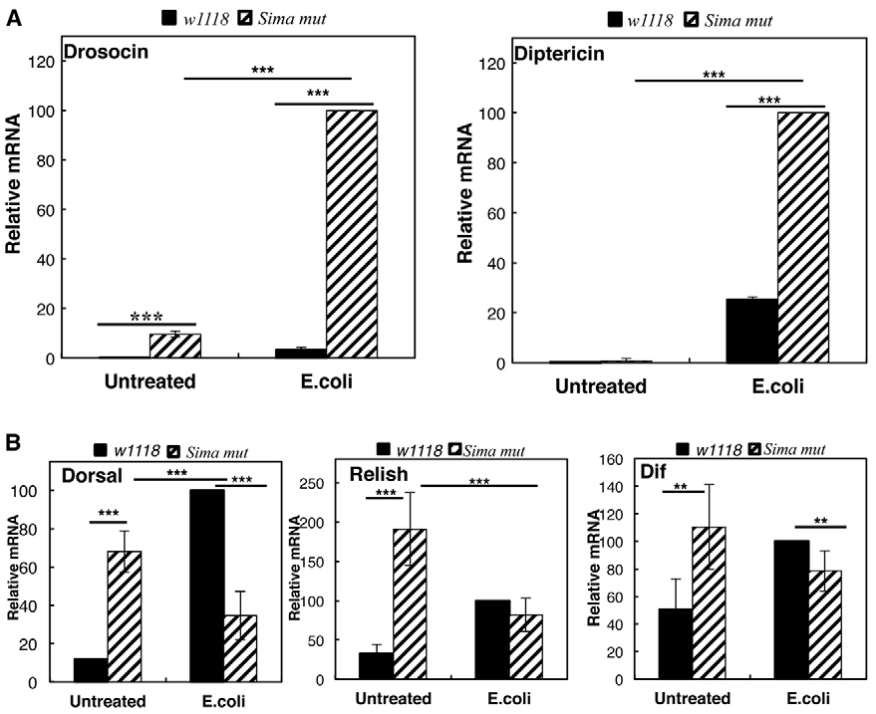
**Role of HIF in the immune response of *Drosophila*.** Third instar larvae were infected with *E. coli* and left for 2 h at 25°C before lysis. Total RNA was extracted from the whole body of control (*w1118*) or HIF-α loss-of-function larvae (*sima^07607^*), and RT-qPCR was performed to determine the levels of *drosocin* and *diptericin* (A) and *dorsal*, *relish and dif* (B) in untreated or infected flies. The graph shows the relative levels of specific mRNA transcripts normalised actin mRNA levels. The means+s.d. were determined from three independent experiments. Student’s *t*-test analysis was performed *P≤0.05, **P≤0.01, ***P≤0.001. See also supplementary material Fig. S2.

All of our analysis so far suggests that, following infection, the HIF pathway is activated, raising the question as to what is the role of HIF in the immune response of *Drosophila*. HIF has been shown to be important in the development and maturation of key innate and adaptive immune mediators, including T-cells, dendritic cells, macrophages and neutrophils in mammalian systems (Gale and Maxwell, 2010; Kojima et al., 2002). The central role of NF-κB in the regulation of this system is well established (Hayden et al., 2006). Both HIF-1α and NF-κB share some target genes of the immune response, including the pro-angiogenic cytokine IL-8 (Maxwell et al., 2007) and the inflammatory regulator TNF-α (Görlach and Bonello, 2008). Based on these observations, it seems likely that HIF could play a supporting role in the NF-κB-led activation of these and other immune-effector genes, acting as a modulator of the immune response. To study the contribution of HIF in the immune response, we used *sima* loss-of-function mutants that have been previously described (Centanin et al., 2005). We confirmed that, in response to hypoxia, *sima* loss-of-function flies no longer activated the HIF-dependent genes *ldh* and *caix* (supplementary material Fig. S2A). Using wild-type and *sima* loss-of-function flies, we analysed the levels of the antibacterial genes *drosocin* and *diptericin* in response to infection with *E. coli* (Fig. 3A). Surprisingly, our analysis revealed that, in the absence of active HIF-α, both *drosocin* and *diptericin* mRNA levels were significantly increased when compared with that of wild-type controls after infection (Fig. 3A). Furthermore, *drosocin* was also increased at basal levels. We also analysed the levels of other NF-κB targets, such as *attacinB* (supplementary material Fig. S2B). Although following infection there was no significant difference between wild-type and *sima* loss-of-function flies, the basal expression levels of the gene encoding the anti-microbial peptide AttacinB were elevated when HIF-α function was impaired. These data suggest a novel role for HIF in the repression of the inflammatory response in *Drosophila*.

In order to investigate whether this enhanced response occurs through NF-κB, we measured the mRNA levels of the genes encoding the NF-κB subunits (*relish*, *dorsal* and *dif*) in the HIF-α mutant flies (Fig. 3B). At the basal level, expression of the NF-κB subunits was increased when compared with that of the wild type, which could explain the increase in the expression of the target genes that we analysed in the HIF mutant flies. However, after infection with *E. coli*, there was a reduction in the levels of the NF-κB subunit genes *dorsal* and *dif* when compared with the wild-type response (Fig. 3B). These results suggest that, in *Drosophila*, HIF-α is involved in the NF-κB response by controlling the levels of the NF-κB subunits and their activity.

### HIF regulation of the NF-κB pathway is conserved in mammalian cells

**Fig. 4.**
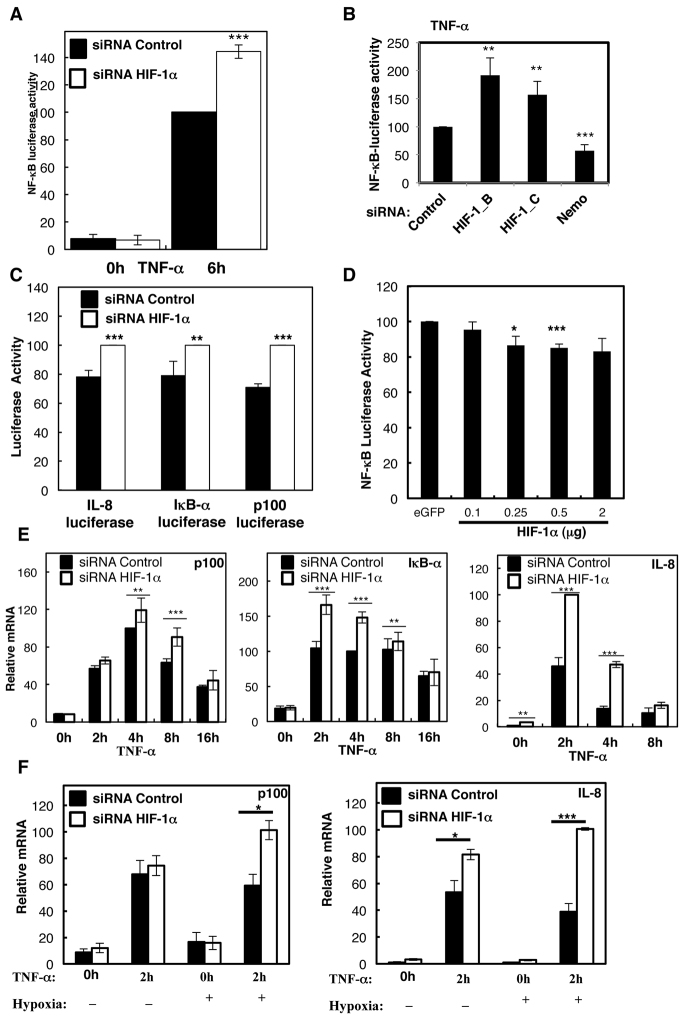
**Regulation of the NF-κB pathway by HIF is conserved in mammalian cells.** (A,B) HeLa cells that had been stably transfected with a κB luciferase reporter were transfected with an siRNA control or siRNA against HIF-1α (with different siRNA oligonucleotides, denoted by B and C) or Nemo. Cells were treated with 10 ng/ml TNF-α for the 6 h prior to luciferase measurements. All the values were normalised to the control with TNF-α. (C) HEK293 cells that had been stably transfected with IL-8, IκB-α and p100 luciferase reporter constructs were transfected with a control siRNA or an siRNA against HIF-1α and then treated with TNF-α for 8 h before luciferase measurements. All the values were normalised to the maximum value of luciferase activity obtained. The graphs depict means+s.d. determined from four independent experiments. Student’s *t*-test analysis was performed *P≤0.05, **P≤0.01, ***P≤0.001. (D) HeLa κB cells were transfected with the indicated concentrations of HIF-α plasmid or empty vector. Cells were treated with TNF-α for 6 h, and the luciferase activity was measured. The values were normalised to the control. (E) HeLa cells were transfected with an siRNA control or an siRNA against HIF-1α. mRNA was extracted, and RT-qPCR was performed for the indicated gene transcripts. The graphs show the relative levels of specific mRNA transcripts normalised to actin mRNA levels. The means+s.d. were determined from four independent experiments. Student’s *t*-test analysis was performed *P≤0.05, **P≤0.01, ***P≤0.001. (F) As in E, but where indicated cells were treated with 1% O_2_ (−hypoxia) for 30 min before the addition of 10 ng/ml TNF-α for an additional 2 h before RNA extraction. See also supplementary material Fig. S3.

The results that we obtained in *Drosophila* suggested that HIF is important in order to constrain the immune response through selective repression of NF-κB. Given the importance of this observation, we decided to investigate whether similar responses exist in mammalian systems. This would also determine the degree of evolutionary conservation in the HIF-mediated repression of the inflammatory response. To address this question, we used siRNA-mediated knockdown of HIF-1α in a mammalian tissue culture model. In mammalian systems, infection with pathogens, such as virus or bacteria, results in the activation of immune cells, such as macrophages. Immune cells release several pro-inflammatory cytokines, including IL-1β and TNF-α (Goldszmid and Trinchieri, 2012). These are potent inducers of NF-κB. To mimic the conditions of inflammation *in vitro*, we used a κB-reporter in HeLa cells to test whether HIF-1α depletion results in changes to NF-κB transcriptional activity following treatment with TNF-α. As observed in flies, HIF-1α knockdown led to a significant increase of the NF-κB activity (Fig. 4A). To rule out off-target effects of the siRNA used, the effects of additional siRNA oligonucleotides targeting HIF-1α were also analysed (Fig. 4B; supplementary material Fig. S3A). This analysis revealed that all of the oligonucleotides tested resulted in a similar de-repression of NF-κB activity (Fig. 4B).

Because the NF-κB activity that we measured was based on an artificial promoter construct, comprising canonical κB sites in tandem, we next investigated a panel of validated NF-κB target gene promoters. The targets and luciferase constructs selected were IL-8, IκB-α and p100 (Fig. 4C). After the addition of TNF-α to the cells for 8 h, all three luciferase constructs, IL-8, IκB-α and p100, presented significantly increased luciferase activity in the HIF-1α knockdown cells when compared with that of the siRNA control (Fig. 4C). This analysis suggests that HIF can repress NF-κB targets in the context of the full promoter.

We also determined whether HIF-1α gain of function can modulate NF-κB activity (Fig. 4D). Overexpression of the HIF-1α construct resulted in a small reduction in NF-κB activity, indicating that increased HIF-1α levels can lead to diminished NF-κB activation. The small effect observed might be derived from compensation mechanisms, as HIF-1α has been shown to regulate the genes encoding cytokines such as TNF-α and IL-1β (Feinman et al., 2010; Fujisaka et al., 2013), two direct activators of NF-κB.

Given our analysis using reporter gene assays, we next determined whether endogenous NF-κB-dependent genes were affected by the decrease of HIF-1α levels. mRNA levels of *p100*, *iκB-α* and *il-8* were monitored in TNF-α-induced HIF-1α knockdown and control cells (Fig. 4E). These experiments demonstrated that, in TNF-α stimulated cells, HIF-1α depletion resulted in increased levels of mRNA encoding IκB-α, p100 and IL-8, in comparison with those of siRNA controls. In addition, the experiments also confirmed the results of the luciferase reporter assays, indicating that HIF-1α depletion increases NF-κB transcriptional activity. We extended our mRNA analysis to a panel of known direct NF-κB target genes (supplementary material Fig. S3B). HIF-1α-mediated repression of NF-κB was also observed when we measured the mRNA and protein levels of A20, Cyld, DDX3, IAP1 and XIAP (supplementary material Fig. S3B), indicating a general mechanism.

As HIF-1α is mainly active in hypoxia, we also performed HIF-1α depletion under conditions of hypoxia, in the presence or absence of TNF-α for 2 h (Fig. 4F). Under these conditions, HIF-1α reduction by using siRNA still resulted in increased levels of NF-κB-dependent targets, such as p100 and IL-8 (Fig. 4F), indicating that HIF-1α-mediated repression of NF-κB is also observed in hypoxic conditions.

### HIF-1α repression of NF-κB requires IKK and TAK1

**Fig. 5.**
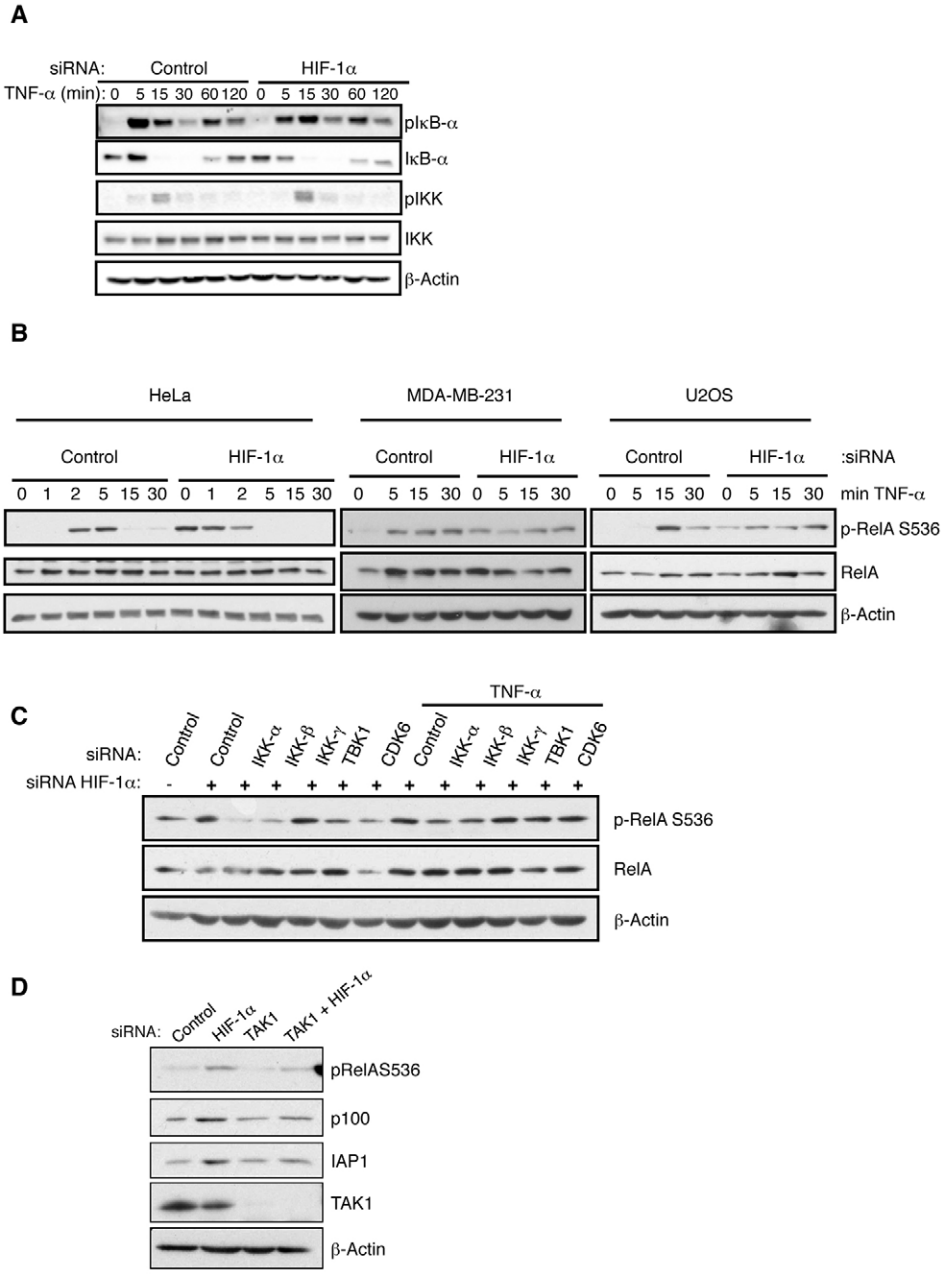
**HIF knockdown induces TAK-IKK activity.** (A) HeLa cells were transfected with an siRNA control or an siRNA against HIF-1α and then treated with 10 ng/ml of TNF-α for the indicated times. Whole-cell lysates were prepared and analysed by western blotting for the indicated proteins (‘p’ prefix denotes phosphorylated protein). (B) HeLa, MDA-MB-231 and U2OS were transfected with an siRNA control or an siRNA against HIF-1α and treated with TNF-α for the indicated times. Whole-cell lysates were prepared and analysed by western blotting for the indicated proteins. (C) HeLa cells were transfected with the indicated siRNA oligonucleotides before treatment with TNF-α for 30 min before harvest. Whole-cell lysates were analysed by western blotting for the depicted proteins. (D) HeLa cells were transfected with the indicated siRNA oligonucleotides before harvest. Whole-cell lysates were analysed by western blotting for the indicated proteins. See also supplementary material Fig. S4.

Our results showed that a reduction of HIF-1α promotes NF-κB transcriptional activity (Fig. 4). Therefore, we decided to investigate the mechanism behind NF-κB repression by HIF-1α. The IKK kinase complex plays a crucial role in NF-κB activation; IKK-dependent phosphorylation of IκB targets IκB for proteasomal degradation and induces NF-κB dimer release. We thus analysed the levels of IKK activation following treatment with TNF-α in the absence or presence of HIF-1α (Fig. 5A). Western blot analysis demonstrated a slightly higher induction of phosphorylation of IKK and a faster degradation of IκB-α in the absence of HIF-1α (Fig. 5A). Interestingly, IKK phosphorylation kinetics did not change in the absence of HIF-1α, suggesting that upstream negative-feedback loops are still in place.

One of the NF-κB subunits, RelA/p65, has been previously shown to be phosphorylated at Ser536 by IKK *in vivo*, resulting in enhanced NF-κB transcriptional activity (Chen et al., 2005; Schmitz et al., 2001). Phosphorylation of Ser536 of RelA has been shown to be important for RelA nuclear import and activity (Perkins, 2012), as well as cancer cell proliferation (Adli and Baldwin, 2006). We next analysed the levels of this phosphorylation following the addition of TNF-α, in the presence or absence of HIF-1α (Fig. 5B). In three different cell lines, TNF-α-induced phosphorylation of p65 at Ser536 was more rapidly induced in the absence of HIF-1α, and furthermore it reached higher levels when compared with that under control conditions (Fig. 5B). Because Ser536 phosphorylation can be mediated by kinases other than IKK (Buss et al., 2004; Buss et al., 2012), we next determined whether IKK was indeed the responsible kinase for the observed enhanced phosphorylation of p65 in the absence of HIF-1α. We depleted all the kinases known to be responsible for this modification on p65 and analysed the Ser536 phosphorylation levels in the presence or absence of HIF-1α (Fig. 5C; supplementary material Fig. S4A). Depletion of CDK6 and TBK1 reduced Ser536 phosphorylation on p65 only in basal conditions following HIF-1α knockdown. Importantly, depletion of IKKα or IKKβ completely reversed the enhanced levels of Ser536 phosphorylation on p65, observed when HIF-1α was depleted (Fig. 5C). Taken together, these data suggest that HIF-1α-mediated repression of NF-κB activity requires the canonical IKKs.

Upstream of the IKK complex lies the TAK-TAB complex (Wuerzberger-Davis and Miyamoto, 2010). Because IKK activation requires TAK1, we next determined whether HIF-1α-mediated repression of NF-κB was dependent on TAK1. To this end, we co-depleted HIF-1α and TAK1, and measured NF-κB transcriptional activity (supplementary material Fig. S5A). Our results show that, in the absence of TAK1, HIF-1α depletion no longer results in increased NF-κB activity. These results are very similar to those obtained using RelA and IKKβ depletion, where HIF-1α knockdown no longer de-repressed NF-κB (supplementary material Fig. S5B,C). To further demonstrate a requirement of TAK1 for HIF-1α-mediated repression of NF-κB, we assessed the levels of p100 and IAP1, two of the NF-κB targets that we analysed above, as well as RelA phosphorylation (Fig. 5D). Co-depletion of HIF-1α and TAK1 completely reversed the levels of p100, IAP1 and phospho-RelA to control levels (Fig. 5D). These results suggest that TAK1 is required for HIF-1α-dependent suppression of NF-κB activity.

### HIF-1α depletion enhances NF-κB activity via a CDK6-dependent mechanism

**Fig. 6.**
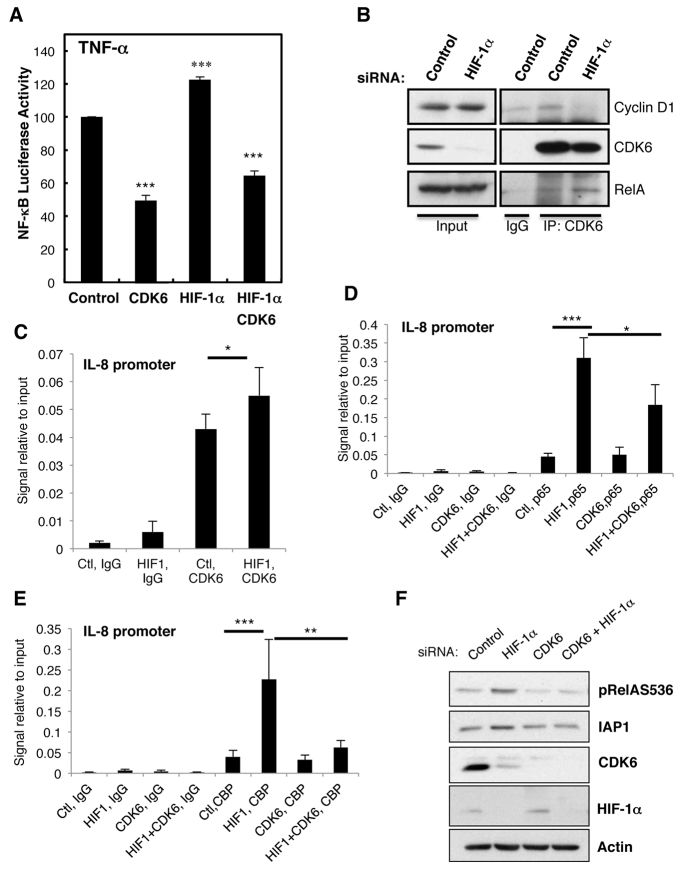
**HIF de-repression of NF-κB activity requires CDK6.** (A) HeLa-κB cells were co-transfected with the indicated siRNA for 48 h. Cells were treated with TNF-α for 6 h, and the luciferase activity was measured. The means+s.d. were determined from three independent experiments. Student’s *t*-test analysis was performed *P≤0.05, **P≤0.01, ***P≤0.001. (B) HeLa cells were transfected with control or HIF-1α siRNA oligonucleotides for 48 h before lysis. To immunoprecipitate CDK6, 200 μg of protein was used. Normal rabbit IgG was used as a control. Immunoprecipitated complexes were analysed by western blotting using the indicated antibodies. Inputs correspond to 10% of material. (C) HeLa cells were transfected with control (Ctl) and HIF-1α (HIF1) siRNA oligonucleotides for 48 h before treatment with 10 ng/ml TNF-α for 1 h. Cells were fixed and lysed, and chromatin immunoprecipitation assays were performed using the indicated antibodies. Purified DNA was analysed by using qPCR with primers for the *IL-8* promoter. The graph depicts means+s.d. from three independent experiments. Student’s *t*-test analysis was performed *P≤0.05, **P≤0.01, ***P≤0.001. (D,E) HeLa cells were transfected with control (Ctl), HIF-1α (HIF1), CDK6 (CDK6) or HIF-1α and CDK6 (HIF1+CDK6) siRNA oligonucleotides for 48 h before treatment with 10 ng/ml TNF-α for 1 h. Cells were processed and analysed as in C. The graph depicts means+s.d. from three independent experiments. Student’s *t*-test analysis was performed *P≤0.05, **P≤0.01, ***P≤0.001. (F) HeLa cells were transfected with control, HIF-1α, CDK6 or HIF-1α and CDK6 siRNA oligonucleotides for 48 h before lysis. Whole-cell lysates were analysed by western blotting for the indicated proteins. See also supplementary material Fig. S5.

A very recent report has demonstrated that NF-κB associates with CDK6 in order to regulate target genes in response to cytokines such as TNF-α (Handschick et al., 2014). Our previous studies have demonstrated that HIF-1α depletion alters the cell cycle through induction of the cyclin-dependent kinase (CDK) inhibitor p21 (Culver et al., 2011). We thus determined whether CDK6 was required for the enhanced NF-κB transcriptional activity that was observed in the absence of HIF-1α (Fig. 6A). Notably, CDK6 depletion completely ablated the effects of the absence of HIF-1α on NF-κB transcriptional activity. This suggests that HIF-1α-dependent suppression of NF-κB transcriptional activity requires CDK6.

To whether HIF-1α depletion alters the association of CDK6 with RelA and/or its canonical binding partner Cyclin D1, we immunoprecipitated CDK6 from cells containing or lacking HIF-1α. Although HIF-1α depletion resulted in a visible decrease of CDK6 levels, the amount of CDK6 recovered following immunoprecipitation was comparable under both conditions tested (Fig. 6B). CDK6 association with Cyclin D1 was not significantly changed, when HIF-1α was depleted, however, CDK6 associated more with RelA. This suggests that HIF-1α depletion promotes CDK6 relocation, into a new complex with RelA.

Next, we investigated by which mechanism CDK6 acts on NF-κB in the absence of HIF-1α. To this end, we analysed whether, following TNF-α stimulation, CDK6 was found at the IL-8 promoter, one of the NF-κB-dependent genes that was most altered in the absence of HIF-1α (see Fig. 4E). We could measure a statistically significant, although modest, increase in the amount of CDK6 that was present at the IL-8 promoter when HIF-1α was depleted (Fig. 6C). Given, this modest effect on CDK6 levels present at the IL-8 promoter, we determined the biological significance of this increased recruitment. For this, we analysed the levels of RelA/p65 occupancy at the IL-8 promoter following TNF-α stimulation upon depletion of HIF-1α, with or without depletion of CDK6 (Fig. 6D). In the absence of HIF-1α, TNF-α induction led to a significant increase in the level of RelA/p65 present at the IL-8 promoter. Importantly, these levels were significantly reduced when HIF-1α was co-depleted with CDK6 (Fig. 6D; supplementary material Fig. S5E). However, the level of RelA/p65 present at this promoter was still higher than when HIF-1α was present, suggesting that CDK6 only partially contributes to this effect of HIF-1α. Because NF-κB transcriptional activity is highly dependent on the recruitment of co-activators to its targets promoters (Perkins, 2006), we determined whether HIF-1α depletion resulted in any changes in the recruitment of CREB-binding protein (CBP) to the IL-8 promoter and whether this was mediated by CDK6 (Fig. 6E; supplementary material Fig. S5E). Our analysis revealed that HIF-1α depletion resulted in increased levels of CBP at the IL-8 promoter when compared with that of control (Fig. 6E). Importantly, this effect was completely abolished when CDK6 was co-depleted with HIF-1α (Fig. 6E), indicating that CDK6 is crucial for the recruitment of co-activators, such as CBP, to the IL-8 promoter when HIF-1α is depleted. Finally, to fully establish the importance of CDK6 in the HIF-1α-mediated effects on NF-κB, we analysed the protein levels of an additional NF-κB target, IAP1, when HIF-1α and CDK6 were co-depleted (Fig. 6F). In the absence of CDK6, HIF-1α depletion was no longer able to increase the levels of IAP1 (Fig. 6F). These results support the idea that HIF-1α-mediated repression of NF-κB activity is facilitated by modulation of the association of CDK6 with RelA at particular target gene promoters.

### Loss of HIF-1α levels results in enhanced angiogenesis in mammalian cells and reduced survival following infection in *Drosophila*

Our results indicate that an important function of HIF-1α is to restrain NF-κB activity. We next wanted to determine the physiological relevance of our mechanistic findings in terms of cell and organism responses.

**Fig. 7.**
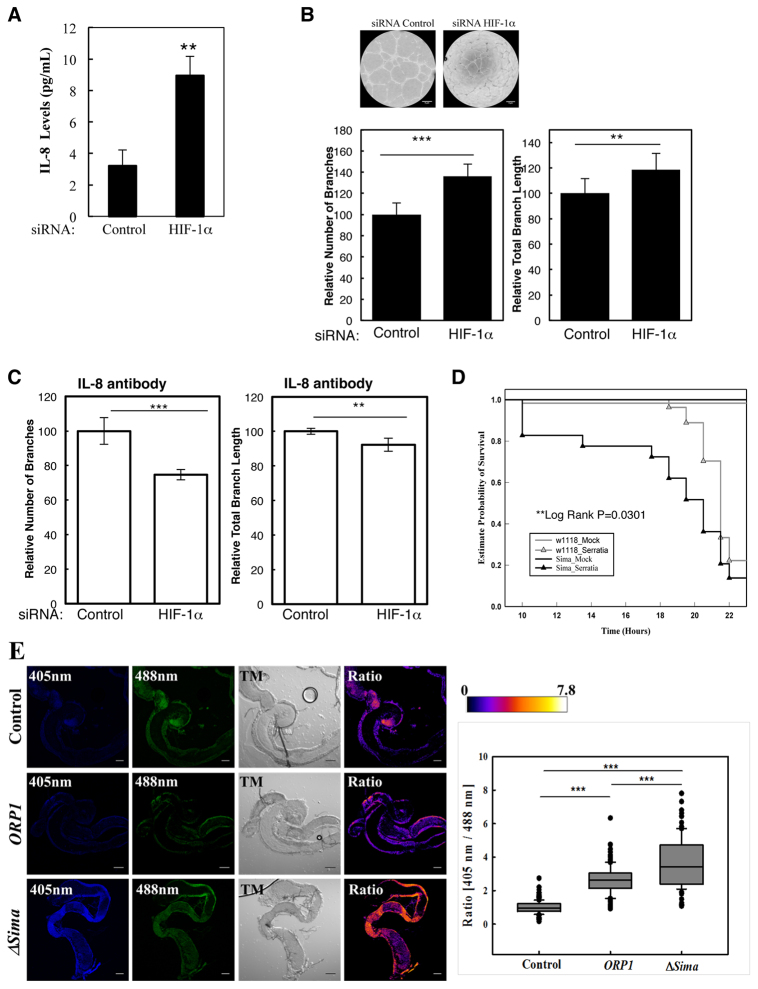
**HIF-1α depletion results in increased angiogenesis in cancer cells and enhanced mortality following infection in *Drosophila*.** (A) IL-8 protein levels were measured in the medium that had been derived from HeLa cells transfected with control or HIF-1α siRNA oligonucleotides for 48 h. The graph depicts the means and standard error of the mean from three independent experiments. (B) HUVEC cells were used to produce endothelial tubes and then incubated with medium derived from HeLa cells transfected with control or HIF-1α siRNA oligonucleotides for 48 h. Example images are shown and the graphs depict mean+s.d. from three independent experiments, where the number of branches and total branch length were measured. Student’s *t*-test analysis was performed *P≤0.05, **P≤0.01, ***P≤0.001. (C) HUVECs were treated as described in B, but medium was incubated with an antibody against IL-8 for 2 h before being used. The graphs depict mean+s.d. from three independent experiments, where the number of branches and total branch length were measured. (D) Wild-type adult flies (*w1118*) and HIF-α loss-of-function flies (*sima^07607^*) were pricked using a thin needle dipped in a diluted overnight culture of *Serratia marcescens* Db10 (OD_600_=0.2) or in a saline solution (Mock). Groups of 60 to 80 flies were used and kept at room temperature. Survival was monitored and expressed as the ‘estimated probability of survival’. The *P*-value was obtained from log-rank statistical analysis. (E) H_2_O_2_ sensing by ICMT flies (Control), roGFP2-ORP1 (ORP1) and Sima loss-of-function roGFP2-ORP1 (ΔSima) transgenic flies was analysed 6 h following infection with *Serratia marcescens*. TM, transillumination or bright field. Images were acquired using a Zeiss confocal microscope and analysed using ImageJ. The ratio image was obtained by dividing the 405-nm image by the 488-nm image pixel by pixel and displayed in false colours using the lookup table Fire (arbitrary units). The values obtained were imported into Sigma-Plot software, which was used to generate box and whisker plots. In the plot, the middle line shows the median value, the bottom and top lines represent the lower and upper quartiles. Whiskers extend to 10th and 90th percentiles and all the outliers are shown. Quantification was performed using ten adult flies in two independent experiments, +s.d. Scale bars: 100 μm. Student’s *t*-test analysis was performed *P≤0.05, **P≤0.01, ***P≤0.001. See also supplementary material Fig. S6.

One of the NF-κB targets that we have analysed is IL-8. IL-8 is an important chemokine that promotes angiogenesis (Waugh and Wilson, 2008). We first measured the levels of IL-8 in the tissue culture medium of cells following depletion of HIF-1α. The analysis revealed that, indeed, IL-8 levels were increased in the absence of HIF-1α (Fig. 7A). We then tested whether the elevation of IL-8 was sufficient to induce changes in an *in vitro* model of angiogenesis, known as tube formation. This assay has been previously shown to respond to changes in IL-8 levels (Chan et al., 2009). We could measure a significant increase in the number of tube branches, as well as in the total length of the branches, when we used medium from HIF-1α depleted cells (Fig. 7B; supplementary material Fig. S6A). Given that there are many potential pro-angiogenic factors in the tissue culture medium, we tested whether we could revert the phenotype observed in the angiogenesis assay by blocking IL-8. To this end, we repeated our analysis but used an IL-8-blocking antibody in the medium. This demonstrated that we could significantly reduce the increase in the number and length of the tube branches (Fig. 7C). This clearly shows that the increased IL-8 levels observed in the absence of HIF-1α will lead to an increased angiogenic response.

Although the response mediated by IL-8 is of great importance, we wanted to analyse the relevance of our findings in the context of a whole organism. To this end, we revisited our *Drosophila* model. Using wild-type and HIF-α (Sima) loss-of-function animals, we analysed the effects of infection with *Serratia marcescens*, a relevant pathogen for *Drosophila* but also for humans (Kounatidis and Ligoxygakis, 2012; Mahlen, 2011). Our analysis revealed that, in the absence of HIF-α, animals were significantly more likely to die from infection than their wild-type counterparts. We obtained similar results with *Drosophila* strains that had either increased NF-κB activity, such as Cyld loss of function, or decreased NF-κB activity, such as IKK/Ird5 loss-of-function flies (supplementary material Fig. S6B,C), indicating that the correct control of NF-κB is crucial for a correct immune system response. Our analysis thus demonstrates that HIF-α is required for proper immune function in the context of a whole organism such as *Drosophila*.

One mechanism by which NF-κB could be activated in the absence of HIF-α is via the increased generation of intracellular reactive oxygen species (ROS) (Gloire et al., 2006). ROS can also lead to HIF-1α stabilisation (Chandel, 2010; Dehne and Brüne, 2014). To determine whether ROS were indeed altered in the absence of HIF-α in *Drosophila*, we took advantage of a H_2_O_2_ biosensor that has been described previously (Albrecht et al., 2011). Analysing the levels of activated sensor following infection with *Serratia marscescens* revealed that, in adult flies with impaired HIF-α function, there was a significant increase in the intracellular levels of H_2_O_2_ detected using this method when compared with that of wild-type flies. These results suggest that ROS generation in the absence of HIF-α are increased in the fly, and this could explain the increased NF-κB activity and, possibly, the loss in viability following infection that was observed in this study.

## DISCUSSION

In this study, we have identified HIF-1α as a repressor of NF-κB activity in cells and in the context of a whole organism, namely *Drosophila melanogaster*. We found that in the absence of HIF-1α/Sima, the levels of several NF-κB targets were elevated, leading to a deregulated immune response in *Drosophila*. In mammalian cells, several NF-κB targets were also elevated in the absence of HIF-1α, in a mechanism that requires TAK-IKK activity. The increased activity of NF-κB is also dependent on CDK6, a kinase that has been recently identified as being important for NF-κB activity at specific promoters (Handschick et al., 2014). Importantly, we demonstrate that depletion of HIF-1α results in an increase in NF-κB-dependent expression of IL-8, which gives rise to an enhanced angiogenesis response. Taken together, these data demonstrate that, in the absence of HIF-1α, NF-κB activity is pathologically deregulated and that this function of HIF-α in restraining NF-κB is evolutionarily conserved.

We, and others, have previously demonstrated that inflammatory cytokines and bacterial products, such as LPS, lead to HIF-1α activation in an NF-κB-dependent manner (Bonello et al., 2007; Rius et al., 2008; van Uden et al., 2008; van Uden et al., 2011). HIF has established roles in the response to hypoxia (Moniz et al., 2014; Semenza, 2011), and several studies using cell-specific HIF-α knockout have established a role for HIFs in the immune response. However, depending on which cell type is analysed, HIF-1α is either pro-inflammatory or anti-inflammatory (reviewed in Scholz and Taylor, 2013). Given the complexity of these systems, we used the *Drosophila* model to investigate the more conserved functions of HIF in the immune response. Our analysis revealed that, just like in mammals, activation of the immune response leads to HIF induction via increased mRNA levels. Our data indicated that this is also NF-κB dependent, as we have previously observed in cell culture mammalian models (van Uden et al., 2008; van Uden et al., 2011). We thus analysed the function of the HIF-α homologue in *Drosophila* in the context of infection. Surprisingly, we found that mutation of *sima* led to a deregulated immune response in adult flies, with increased levels of antimicrobial peptide transcription, as well as increased NF-κB activity. This suggests that HIF-α, in *Drosophila*, functions to restrain inflammation and control the innate immune response. Importantly, inhibition of HIF-α in the fly makes the organism susceptible to death following infection, suggesting that the NF-κB-dependent defence against pathogens requires negative-feedback mechanisms to provide appropriate levels of immune response. A similar phenotype has been reported for the NF-κB negative regulator Cyld (Bandarra et al., 2014; Tsichritzis et al., 2007). Loss-of-function strains for *Cyld* were found to have reduced viability in response to infection (Tsichritzis et al., 2007) and enhanced NF-κB activity (Bandarra et al., 2014). However, because both HIF and Cyld have additional functions in the cell, it is also possible that the reduced viability might be owing to factors other than NF-κB. One possibility is the increase in ROS that was detected following infection in the strains lacking functional HIF-α. Although ROS can also lead to increased NF-κB activation (Gloire et al., 2006), it is possible that, by itself, ROS can reduce the viability of the animals. Interestingly, the use of anti-oxidants, such as ascorbic acid, has been shown to block cancer-associated inflammation, namely sepsis (Ichim et al., 2011). In *Drosophila* and also in mammalians systems, HIF-1α is known to activate the expression of REDD1 (Shoshani et al., 2002). Recently, REDD1 has been shown to be crucial for the control of ROS production in hypoxia (Horak et al., 2010). Furthermore, other studies have demonstrated that mammalian HIF-1α is required to control toxic ROS production in hypoxia, partially via activation of pyruvate dehydrogenase kinase 1 (Kim et al., 2006). Additional work would be necessary to fully investigate this phenotype.

We have found *sima* loss-of-function flies to be more susceptible to infection by *Serratia marcescens* and *Escherichia coli*. Although these are both Gram-negative bacteria, they seem to be differentially susceptible to anti-bacterial peptides in *Drosophila*. Although Drosocin and Diptericin are able to kill *Escherichia coli*, no activity towards *Serratia marcescens* has been reported (Bikker et al., 2006; Cudic et al., 1999). This might explain the difference in the timing of *Drosophila* death observed following infection with the two bacterial strains used.

Although it is well established that NF-κB controls HIF, very little information is available for the control of NF-κB by HIFs. Previous results in neutrophils have shown that hypoxia induced neutrophil survival via HIF-1α-mediated control of NF-κB activity (Walmsley et al., 2005). Our analysis, using several mammalian cancer cells, demonstrated that HIF-α suppressed NF-κB activity in response to a known inducer, TNF-α. Although some genes were also altered at basal levels, the majority of the NF-κB-dependent genes that we have measured are increased in the absence of HIF-1α in response to TNF-α. However, despite this enhanced level of expression, the kinetics of the response to TNF-α appeared unaltered, indicating that termination of the NF-κB response still occurs. The importance of controlling NF-κB is exemplified by the many negative-feedback mechanisms that cells have in place (Renner and Schmitz, 2009; Terry and Chaplain, 2011). In fact, the NF-κB pathway has been the subject of intense mathematical modelling (Ashall et al., 2009; Sung et al., 2014; Terry and Chaplain, 2011). Because termination of the NF-κB response still occurs in the absence of HIF-α, it suggests that all these negative-feedback points are still in place. As such, we observed no significant reduction in the levels of IκBα, A20 or Cyld. In fact, many of these were increased at the transcription level because these are also NF-κB targets (Ashall et al., 2009). It would, therefore, be of interest to consider additional NF-κB negative regulators, in the form of HIF-1α, in mathematical models of NF-κB activation.

Mechanistically we have identified involvement of both TAK1 and IKK, both of which are also present in *Drosophila* (Kleino and Silverman, 2014). These enzymes are required for TNF-α-mediated activation of NF-κB, and as such their involvement in the conditions we have analysed is unsurprising. We have mainly analysed the response to TNF-α, which in *Drosophila* is similar to the immune deficiency (IMD) pathway. However, some of the targets we have analysed, such as *diptericin*, can be synergistically activated by a combination of both Toll and IMD pathways (Tanji et al., 2007). This suggests that both pathways could be altered in *sima* loss-of-function strains. Additional work would therefore be necessary to determine whether any of the components of the Toll pathway in *Drosophila* are regulated by Sima in normoxia or hypoxia.

In the mammalian system, we have identified CDK6 as being required for the HIF-1α-mediated effect on NF-κB. Our previous studies have shown that HIF-1α depletion results in increases in p21 (Culver et al., 2011). p21 is a well-known CDK inhibitor, and in particular, it inhibits canonical CDK activity and promotes cell cycle arrest (Yoon et al., 2012). CDK6 and p21 have both been shown to induce NF-κB activity (Buss et al., 2012; Perkins et al., 1997). CDK6 can phosphorylate RelA at residue Ser536 (Buss et al., 2012) and more recently has been shown to associate with RelA at promoters (Handschick et al., 2014). The relationship between p21 and RelA has been documented via its role in regulating the RelA association with co-activators and the induction of NF-κB transcription activity (Perkins et al., 1997; Poole et al., 2004). Our data are consistent with a role for CDK6 in mediating HIF-1α repression of RelA. Our data indicates that HIF-1α depletion results in an increased association of RelA with CDK6. Although p21 is known to inhibit CDK6 activity, CDK6 action on p65-dependent promoters does not seem to require kinase activity (Handschick et al., 2014). It is thus possible that, through the action of p21, CDK6 is able to associate more with RelA, when HIF-1α is depleted. Further analysis is therefore needed to assess whether this is the case.

Taken together, our data indicates that HIF-1α function in response to infection and inflammation is to restrain NF-κB activity, thus preventing excessive and harmful pro-inflammatory responses.

## MATERIALS AND METHODS

### Cells

Human embryonic kidney (HEK293), human cervix carcinoma (HeLa), human breast adenocarcinoma (MDA-MB-231) and human osteosarcoma (U2OS) cell lines were obtained from the American Type Culture Collection (ATCC). All cells were maintained under 5% CO_2_ in Dulbecco’s modified Eagle’s medium (Lonza) supplemented with 10% fetal bovine serum (FBS) (Invitrogen), 1% penicillin-streptomycin (Lonza) and 1% L-glutamine (Lonza). HeLa-κB luciferase cells were a kind gift from Ron Hay (College of Life Sciences, University of Dundee, Dundee, UK) and have been described previously (Campbell et al., 2006). Human umbilical vein endothelial cells (HUVECs) were obtained from Life Technologies and cultured in medium 200PRF (Invitrogen) with low serum growth supplement (Invitrogen) and 1% penicillin-streptomycin (Lonza).

### Treatments

TNF-α was obtained from Peprotech, dissolved in PBS and used at a final concentration of 10 ng/ml, subsequent to medium change. Hypoxia treatments were performed in an InVIVO 300 hypoxia workstation (Ruskinn).

### Plasmids

IL-8 luciferase has been described previously (Culver et al., 2010). p100 and IκB-α luciferase constructs were a kind gift from Neil Perkins (Newcastle University, Newcastle, UK) and Ron Hay, respectively.

### siRNA

siRNA oligonucleotides were purchased from MWG and used at a final concentration of 27 nM. They were transfected using INTERFERin from Polyplus according to manufacturer’s instructions. The following oligonucleotide sequences were used for siRNA knockdown: control, 5′-AACAGUCGCGUUUGCGACUGG-3′; HIF-1α, 5′-CUGAUGACCAGCAACUU-3′; HIF-1α_B, 5′-GGAAUUGGAACAUUAUUAC-3′; HIF-1α_C, 5′-GCAUAUAUCUAGAAGGUAU-3′; HIF-2α, 5′-CAGCAUCUUUGACAG-3′; TBK1, 5′-GACAGAAGUUGUGAUCACA-3′; CDK6 5′-GAAGACUGGCCUAGAGAUG-3′. siRNAs against IKKα, IKKβ, IKKγ and RelA have been described previously by Kenneth et al. (Kenneth et al., 2010), and against p21 by Yamada et al. (Yamada et al., 2013).

### Antibodies

Antibodies against p21 and HIF-1α were from BD Biosciences; p-RelA, pIKK, pIκB-α, IKK, IκB-α and β-actin were from Cell Signaling; IL-8 from R&D systems; CDK6 and RelA from Santa Cruz Biotechnology. Antibodies against Relish, Dorsal, Cactus, α-Tubulin and Tango were from Developmental Studies Hybridoma Bank (DSHB). Enhanced chemiluminescence (Pierce) was used for detection.

### *Drosophila* strains, RNA and protein extract preparation

Fly culture and husbandry were performed according to standard protocols. Homozygous flies carrying the *ird5^1^* mutation (Lu et al., 2001) were used as an IKK loss-of-function model, and *sima^07607^* (Centanin et al., 2005) flies were used as a HIF-1α loss-of-function model. *white^1118^* flies were used as control. H_2_O_2_ sensor strains were obtained from Tobias Dick (Heidelberg, Germany) (Albrecht et al., 2011) and crossed into the *white^1118^* or *sima^07607^* background. The *dipt2-2-LacZ* strain (Benassi et al., 2000) was obtained from Dominique Ferrandon (Strasbourg, France). Homozygous flies carrying *cyldf00814* mutation were used as CYLD loss-of-function (Tsichritzis et al., 2007).

Third instar larvae or adults were pricked with a Tungsten needle inoculated with a concentrated bacterial solution (OD_600_≈200) from an overnight culture of *E. coli* and incubated for 2 h at 25°C. For protein extracts, adult heads were collected from ~30 adult flies aged 3–5 days, homogenized in 100 μl of RIPA buffer (50 mM Tris pH 8, 150 mM NaCl, 1% Igepal, 0.5% sodium deoxycholate, 0.1% SDS) and then centrifuged at 10,000 ***g*** at 4°C for 10 min. mRNA extracts were prepared from adult heads or whole-larvae body using TRIzol (Invitrogen). Gene expression levels of *diptericin*, *drosomycin*, *sima*, *tango*, *drosocin*, *ldh*, *fatiga*, *caix*, *relish*, *dorsal* and *dif* were measured by using qRT-PCR. The following primers were used for qRT-PCR: *Drosophila* Actin, F: 5′-GCGTTTTGTACAATTCGTCAGCAACC-3′, R: 5′-GCACGCGAAACTGCAGCCAA-3′. Sima, F: 5′-AGCCCAATCTGCCGCCAACC-3′, R: 5′-TGGAGGCCAGGTGGTGGGAC-3′. Tango, F: 5′-CGGCTGCTCATACGCCCGAG-3′, R: 5′-TGGAGGCCAGGTGGTGGGAC-3′. Fatiga, F: 5′-TGGCCCGCCGAGGTAGACAA-3′, R: 5′-CAGGCCCGTCTCCATCCCCA-3′. *Drosophila* Ldh, F: 5′-CAGTTCGCAACGAACGCGCA-3′, R: 5′-CAGCTCGCCCTGCAGCTTGT-3′. Diptericin, F: 5′-ACCGCAGTACCCACTCAATC-3′, R: 5′-ACTTTCCAGCTCGGTTCTGA-3′. Drosocin, F: 5′-CCATCGTTTTCCTGCTGCTTGC-3′, R: 5′-GTCAGGTGATCCTCGATGGCCA-3′. Drosomycin, F: 5′-GTTCGCCCTCTTCGCTGTCCTGA-3′, R: 5′-CCTCCTCCTTGCACACACGACG-3′. *Drosophila* CAIX, F: 5′-GGGCCCGCCATCATGGTCAC-3′, R: 5′-AATGGCCGTTGTCCGGCTGG-3′. Relish, F: 5′-GACCCGAAAGCTCGGCGCAAA-3′, R: 5′-TCGCTCACGAGTTGCGAGCAA-3′. Dorsal, F: 5′-TGTTCAAATCGCGGGCGTCGA-3′, R: 5′-TCGGACACCTTCGAGCTCCAGAA-3′. Dif, F: 5′-CGGACGTGAAGCGCCGACTTG-3′, R: 5′-CAGCCGCCTGTTTAGAGCGG-3′. Attacin B, F: 5′-GGGTAATATTTAACCGAAGT-3′, R: 5′-GTGCTAATCTCTGGTCATC-3′.

### mRNA extract preparation and quantitative PCR analysis

Total RNA from mammalian cells was extracted using peqGOLD total RNA kit (Peqlab) according to the manufacturer’s directions. RNA was converted to cDNA using Quantitect Reverse Transcription Kit (Qiagen). For quantitative PCR, Brilliant II Sybr green kit (Stratagene/Agilent), including specific MX3005P 96-well semi-skirted plates, was used to analyse samples on the MX3005P qPCR platform (Stratagene/Agilent). Actin was used as a normalising gene in all experiments. The following primers were used for RT-qPCR: actin, F: 5′-CTGGGAGTGGGTGGAGGC-3′, R: 5′-TCAACTGGTCTCAAGTCAGTG-3′. p100, F: 5′-AGCCTGGTAGACACGTACCG-3′, R: 5′-CCGTACGCACTGTCTTCCTT-3′. IL-8, F: 5′-CCAGGAAGAAACCACCGGA-3′, R: 5′-GAAATCAGGAAGGCTGCCAAG-3′. XIAP, F: 5′-CTGTTAAAAGTCATCTTCTCTTGAAA-3′, R: 5′-GACCCTCCCCTTGGACC-3′. HIF-1α, F: 5′-CATAAAGTCTGCAACATGGAAGGT-3′, R: 5′-ATTTGATGGGTGAGGAATGGGTT-3′. A20, F: 5′-ACAGCTTTCCGCATATTGCT-3′, R: 5′-TTGACCAGGACTTGGGACTT-3′. IAP1, F: 5′-AACTCTTGGCCTTTCATTCG-3′, R: 5′-TGTTGTGATGGTGGCTTGAG-3′. IκB-α, F: 5′-AAAGCCAGGTCTCCCTTCAC-3′, R: 5′-CAGCAGCTCACCGAGGAC-3′.

Primer sets for *CYLD* and *DDX3* were obtained from Qiagen.

### Whole-cell protein lysates and western blotting

Whole-cell protein lysates were obtained and western blotting was performed, essentially, as described previously (van Uden et al., 2011).

### Chromatin immunoprecipitation

Proteins were cross-linked with formaldehyde for 10 min. Then 0.125 mol/l glycine was added, and cells were washed with PBS. Cells were lysed with lysis buffer (1% SDS, 10 mM EDTA, 50 mM Tris-HCl, pH 8.1, 1 mM PMSF, 1 mg/ml leupeptin, 1 mg/ml aprotonin), followed by sonication and centrifugation. The supernatant was pre-cleared with sheared salmon sperm DNA and protein G Sepharose beads (Sigma). The supernatant was incubated with specific antibodies overnight, and then with protein G Sepharose beads for 1 h. After an extensive wash step, the complexes were eluted with buffer (100 mmol/l NaHCO_3_, 1% SDS) and incubated with proteinase K. DNA was purified using QIAquick polymerase chain reaction purification kit (Qiagen). PCR was performed using the primers for the *IL-8* promoter as described previously (Culver et al., 2010).

### Angiogenesis

Angiogenesis assays were performed using HUVECs and μ-slides (Ibidi). Briefly, 10 μL of Geltrex (Invitrogen) was added to the inner well and allowed to set at 37°C. Then 6×10^5^ HUVECs were suspended in 300 μl of medium comprising one third complete Medium200PRF and two thirds conditioned medium from assay condition. Of this mixture, 50 μl containing 1×10^5^ cells was plated in the outer well of the μ-slide. Cells were allowed organise on the recombinant basement membrane during incubation overnight at 37°C before images were acquired by using microscopy. The number and length of tube branches were calculated using the images acquired with ImageJ software (National Institutes of Health).

### ELISA assay

IL-8 protein levels were measured in the medium from HeLa cells using an ELISA cytokine assay from Qiagen, according to manufacturer’s instructions.

### H_2_O_2_ ROS analysis in *Drosophila*

H_2_O_2_ quantification was achieved by following a method that has been described previously (Albrecht et al., 2011) with slight modifications. Briefly, adult flies were pricked with a Tungsten needle that had been inoculated with a concentrated bacterial solution (OD_600_≈200) from an overnight culture of *Serratia marcescens*, the flies were then incubated for 6 h at 25°C. Flies were then dissected, the abdomen cut and opened in the presence of 20 mM N-ethylmaleimide. Samples were then fixed with 4% PFA for 15 min at room temperature. Fixed tissues were imaged with a Zeiss LSM 700 confocal microscope with a 10× objective. Image processing was performed as described previously (Albrecht et al., 2011).

### Additional experimental procedures

Additional experimental procedures, such as immunoprecipitations and luciferase assays, have been described previously (Kenneth et al., 2013).

## Supplementary Material

Supplementary Material
