# Supplementary material for: HIF-1α restricts NF-κB-dependent gene expression to control innate immunity signals
Source: Dis Model Mech. 2014 Dec 15;8(2):169–81. doi: 10.1242/dmm.017285 (PMC4314782; doi:10.1242/dmm.017285)
Supplement: Supplementary Material [file supp_8_2_169__index.html]

HIF-1α restricts NF-κB-dependent gene expression to control innate immunity signals — Supplementary Material 

# HIF-1α restricts NF-κB-dependent gene expression to control innate immunity signals

## DMM017285 Supplementary Material

**Files in this Data Supplement:**

- **Supplementary Material**
