# Supplementary material for: HIF-1α restricts NF-κB-dependent gene expression to control innate immunity signals
Source: Dis Model Mech. 2014 Dec 15;8(2):169–81. doi: 10.1242/dmm.017285 (PMC4314782; doi:10.1242/dmm.017285)
Supplement: Supplementary Material [file supp_8.2.169_DMM017285.pdf]

Supplementary Information

Supplementary Figure Legends

Figure S1. ***drosomycin*** **induction following** ***E. coli*** **infection is Ird5 dependent**. Third instar larvae were infected with *E. coli* and left for 2h at 25C prior to lysis. Total RNA was extracted from the full body of control larvae (*w1118*), or IKK loss-of-function (Δ*Ird5*), and RT-qPCR was performed for the levels of *Drosomycin* in untreated or infected flies. The graph shows relative levels of specific mRNA transcripts normalized to actin mRNA levels. The mean + SD were determined from three independent experiments. Student t-test analysis was performed * $P \leq 0.05$, ** $P \leq 0.01$, *** $P \leq 0.001$. Related to Figure 2.

Figure S2. ***sima*** **loss-of-function flies do not activate Sima-dependent genes in hypoxia.** (A) Third instar larvae were exposed to 5% O2 for 24h at 25C prior to lysis. Total RNA was extracted from the full body of control larvae (*w1118*), or HIF-α loss-of-function ($sima^{07607}$), and RT-qPCR was performed for the levels of *caix*, *ldha*, (B) Third instar larvae were infected with *E. coli* and left for 2h at 25C prior to lysis. Total RNA was extracted from the full body of control larvae (*w1118*), or HIF-α loss-of-function ($sima^{07607}$), and RT-qPCR was performed for the levels of *attacinB* in untreated or infected flies. The graph shows relative levels of specific mRNA transcripts normalized to actin mRNA levels. The mean + SD were determined from three independent experiments. Student t-test analysis was performed * $P \leq 0.05$, ** $P \leq 0.01$, *** $P \leq 0.001$. Related to Figure 3.

Figure S3. HIF-1α depletion in mammalian cells results in increased NF-κB targets. (A) HeLa cells stably transfected with κB luciferase reporter were transfected with siRNA control, HIF-1α (with different siRNA oligonucleotides), for 48h prior to lysis. Cells were treated with 200μM DFX for the 3h where indicated. Whole cell lysates were analysed by western blot using the indicated antibodies. (B) HeLa cells were transfected with siRNA control or HIF-1α. mRNA was extracted, and RT-qPCR was performed for the indicated gene transcripts. The graphs show relative levels of specific mRNA transcripts normalized to actin mRNA levels. The mean + SD were determined from four independent experiments. Student t-test analysis was performed * $P \leq 0.05$, ** $P \leq 0.01$, *** $P \leq 0.001$. Related to Figure 4.

Figure S4. Validation of p65-regulating kinase depletion by siRNA. HeLa cells were transfected with the depicted siRNA oligonucleotides for 48h prior to lysis. Whole cell lysates were prepared and analysed by western blot for the indicated proteins. Related to Figure 5.

Figure S5. HIF de-repression of NF-κB requires IKK and TAK1 (A-C) HeLa κB cells were co-transfected with the indicated siRNA for 48h. Cells were treated with TNF-α for 6h and luciferase activity measured. The mean + S.D. were determined from three independent experiments. Student t-test analysis was performed * $P \leq 0.05$, ** $P \leq 0.01$, *** $P \leq 0.001$. (D) HeLa cells were transfected with control (Ctl), HIF-1α (HIF1), CDK6 (CDK6) or HIF-1α and CDK6 (HIF1+CDK6) siRNA oligonucleotides for 48h prior to treatment with 10ng/mL TNF-α for 1h. Cells were fixed, lysed and ChIPs were performed using the indicated antibodies. Purified DNA

was analysed by qPCR using primers for the IL-8 promoter. Graph depicts mean + S.D. from three independent experiments. Related to Figure 6.

Figure S6. HIF-1α depletion results in increased angiogenesis in cancer cells and deregulated NF-κB results in increased mortality following infection in *Drosophila*. (A) HUVEC cells were used to produce endothelial tubes and incubated with media derived from HeLa cells transfected with control or HIF-1α siRNA oligonucleotides for 48h and treated or not with TNF-α for 24h. Image is a representative from three independent experiments. (B) Wildtype adult flies (*w1118*), and CYLD loss-of-function flies were pricked using a thin needle dipped in a diluted overnight culture of *Serratia marcescens* Db10 (OD_{600}=0.2) or in a saline solution (Mock). Groups of 60 to 80 flies were used, and kept at room temperature. Survival was monitored and expressed as Estimated probability of Survival. *P*-value was obtained from Log-Rank statistical analysis. (C) Wildtype adult flies (*w1118*), and IKK loss-of-function flies (*Ird5*) were pricked using a thin needle dipped in a diluted overnight culture of *Serratia marcescens* Db10 (OD_{600}=0.2) or in a saline solution (Mock). Groups of 60 to 80 flies were used, and kept at room temperature. Survival was monitored and expressed as Estimated probability of Survival. *P*-value was obtained from Log-Rank statistical analysis. Related to Figure 7.

Figure S1

A

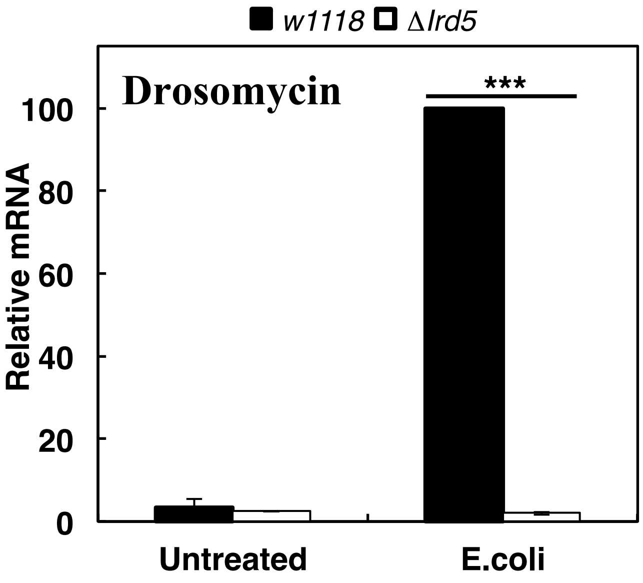
w1118
ΔIrd5
Drosomycin
***
Relative mRNA
100
80
60
40
20
0
Untreated
E.coli

Figure S2

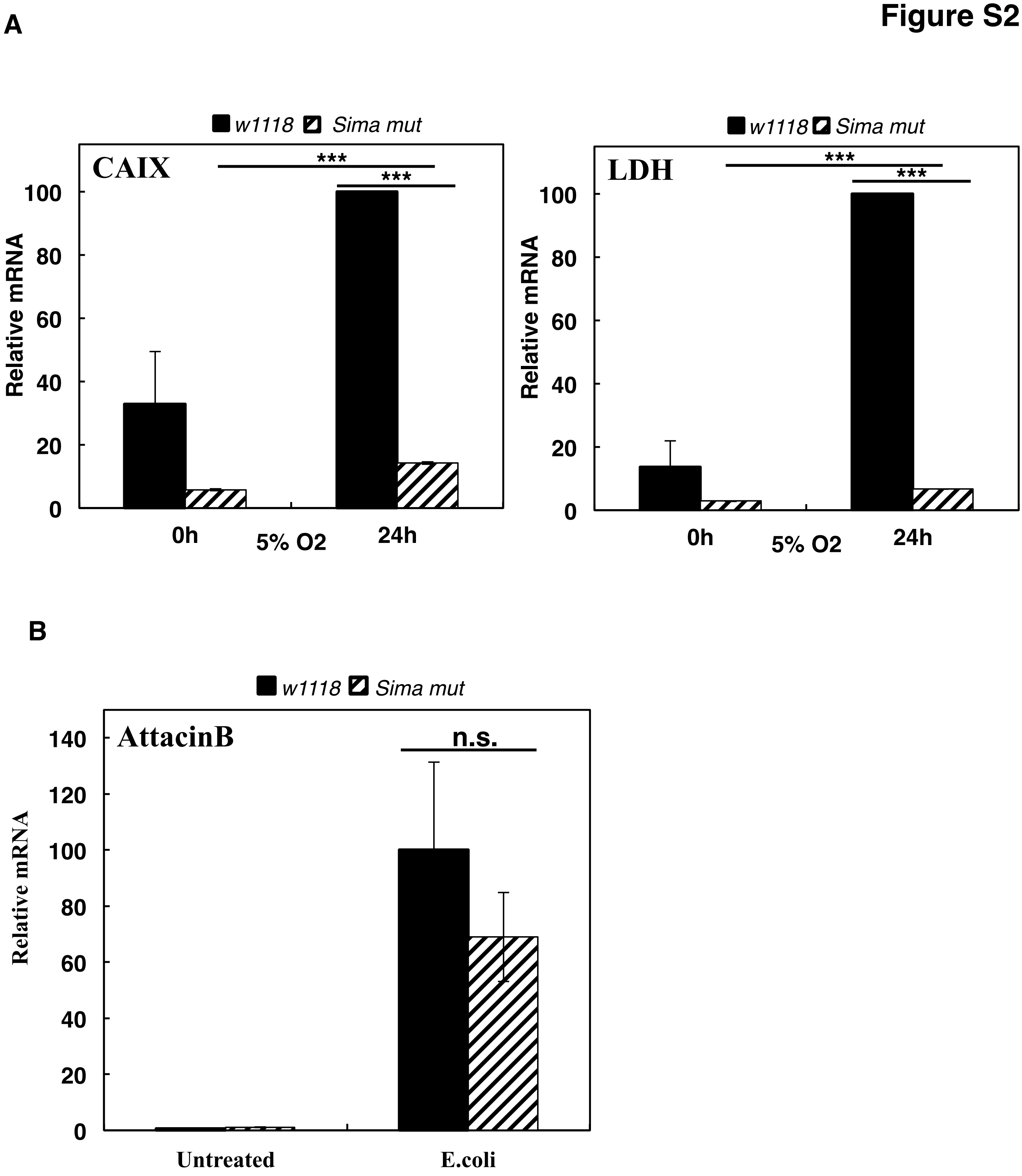
A
w1118
Sima mut
CAIX
***
***
Relative mRNA
100
80
60
40
20
0
0h
5% O2
24h
w1118
Sima mut
LDH
***
***
Relative mRNA
100
80
60
40
20
0
0h
5% O2
24h
B
w1118
Sima mut
AttacinB
n.s.
Relative mRNA
140
120
100
80
60
40
20
0
Untreated
E.coli

Figure S3

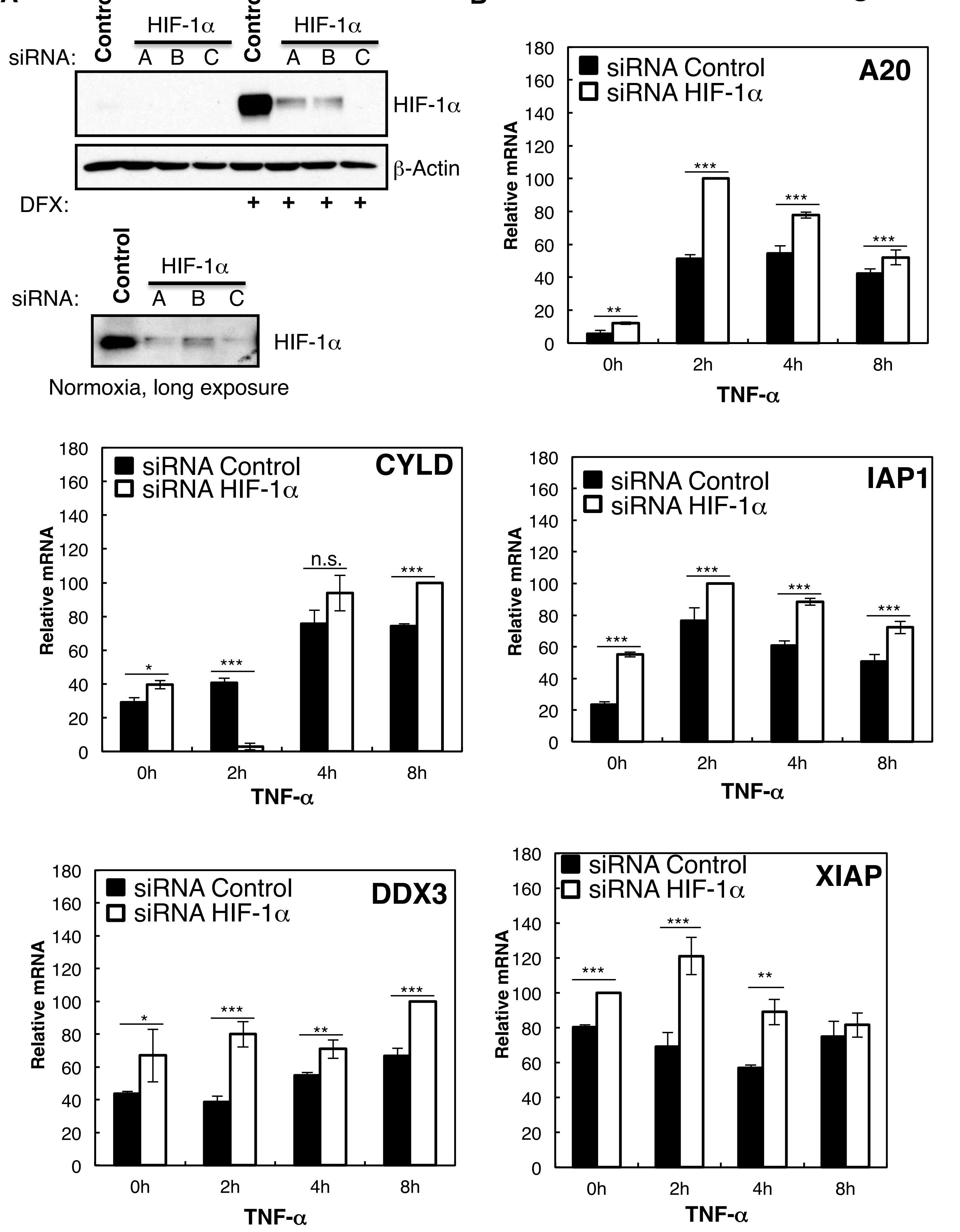
A
siRNA:
Control
HIF-1α
A B C
Control
HIF-1α
A B C
HIF-1α
β-Actin
DFX:
+ + + +
siRNA:
Control
HIF-1α
A B C
HIF-1α
Normoxia, long exposure
B
A20
CYLD
IAP1
DDX3
XIAP
siRNA Control
siRNA HIF-1α
Relative mRNA
0h
2h
4h
8h
TNF-α
n.s.

Figure S4

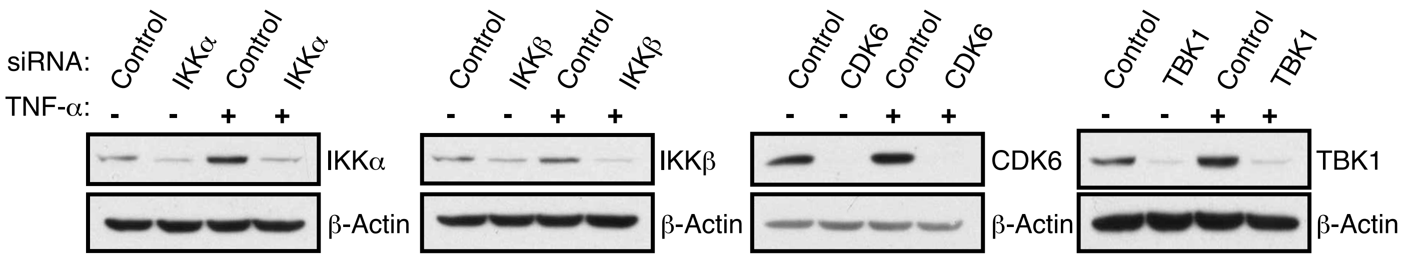
siRNA:
TNF-α:
Control
IKKα
Control
IKKα
- - + +
IKKα
β-Actin
Control
IKKβ
Control
IKKβ
- - + +
IKKβ
β-Actin
Control
CDK6
Control
CDK6
- - + +
CDK6
β-Actin
Control
TBK1
Control
TBK1
- - + +
TBK1
β-Actin

Figure S5

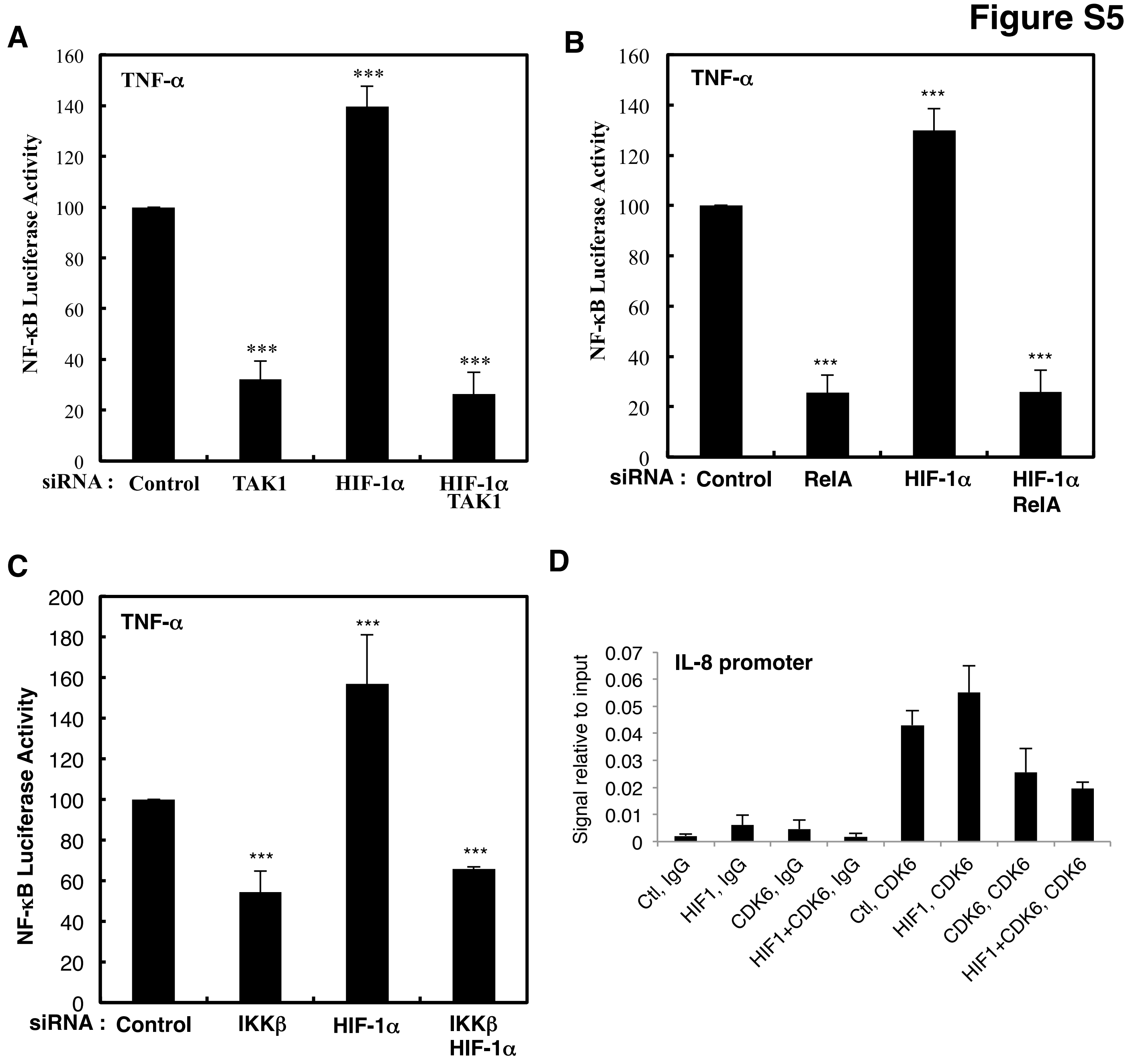
A
TNF-α
NF-κB Luciferase Activity
siRNA : Control TAK1 HIF-1α HIF-1α TAK1
B
TNF-α
NF-κB Luciferase Activity
siRNA : Control RelA HIF-1α HIF-1α RelA
C
TNF-α
NF-κB Luciferase Activity
siRNA : Control IKKβ HIF-1α IKKβ HIF-1α
D
IL-8 promoter
Signal relative to input
Ctl, IgG
HIF1, IgG
CDK6, IgG
HIF1+CDK6, IgG
Ctl, CDK6
HIF1, CDK6
CDK6, CDK6
HIF1+CDK6, CDK6

Figure S6

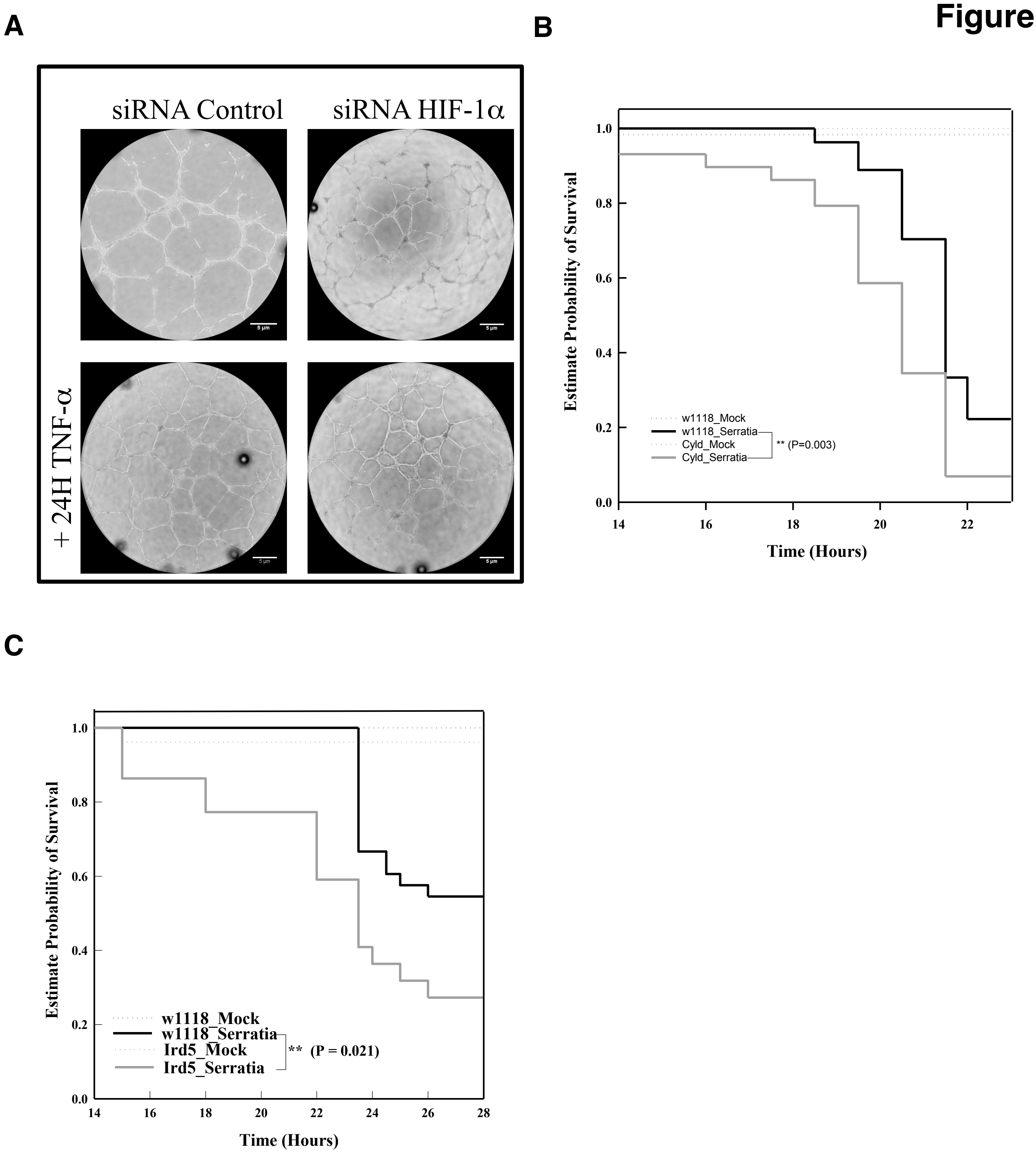
A
siRNA Control
siRNA HIF-1α
+ 24H TNF-α
B
Estimate Probability of Survival
Time (Hours)
w1118_Mock
w1118_Serratia
Cyld_Mock
Cyld_Serratia
** (P=0.003)
C
Estimate Probability of Survival
Time (Hours)
w1118_Mock
w1118_Serratia
Ird5_Mock
Ird5_Serratia
** (P = 0.021)
